# Using automated pharmacy records to assess the management of tuberculosis.

**DOI:** 10.3201/eid0506.990607

**Published:** 1999

**Authors:** G S Subramanyan, D S Yokoe, S Sharnprapai, E Nardell, E McCray, R Platt

**Affiliations:** Brigham and Women's Hospital, Boston, Massachusetts, USA.

## Abstract

We used automated pharmacy dispensing data to characterize tuberculosis (TB) management for 45 health maintenance organization (HMO) members. Pharmacy records distinguished patients treated in HMOs from those treated elsewhere. For cases treated in HMOs, they provided useful information about appropriateness of prescribed regimens and adherence to therapy.


### Using Automated Pharmacy Records to Assess the Management of Tuberculosis

**Girish S. Subramanyan,* Deborah S. Yokoe,* Sharon Sharnprapai,† Edward Nardell,† Eugene McCray,‡ and Richard Platt*§**
*Brigham and Women's Hospital, Boston, Massachusetts, USA; †Massachusetts Department of Public Health, Jamaica Plain, Massachusetts, USA; ‡Centers for Disease Control and Prevention, Atlanta, Georgia, USA; §Harvard Medical School, Harvard Pilgrim Health Care, Boston, Massachusetts, USA

We used automated pharmacy dispensing data to characterize tuberculosis (TB) management for 45 health maintenance organization (HMO) members. Pharmacy records distinguished patients treated in HMOs from those treated elsewhere. For cases treated in HMOs, they provided useful information about appropriateness of prescribed regimens and adherence to therapy.

Health-care coverage, especially from health maintenance organizations (HMOs), often includes pharmacy benefits. Pharmacy dispensing records can identify cases of tuberculosis (TB) unknown to the public health system (1). In this article, we examine the utility of automated pharmacy dispensing data in assessing the quality of management of active TB and patients' compliance with recommended therapy.

### Methods

We used automated pharmacy dispensing records to characterize therapy in 45 cases of active TB diagnosed from January 1, 1992, through June 30, 1996, at Harvard Pilgrim Health Care, a mixed model HMO in New England. These were all known cases of TB in a sample of 350,000 HMO members (1,2); all met the Centers for Disease Control and Prevention's (CDC) surveillance case definition (3). Cases were drawn from the 90% of HMO members with pharmacy benefits.

We identified all dispensings of isoniazid, rifampin, pyrazinamide, ethambutol, streptomycin, ethionamide, kanamycin, cycloserine, capreomycin, and para-aminosalicylic acid (PAS). We also reviewed the full medical records. Empiric regimens, i.e., those dispensed before susceptibility results were known, and final treatment regimens were graded for consistency with American Thoracic Society (ATS) and CDC guidelines in effect at the time of diagnosis (4,5). Two measures of the adequacy of therapy were calculated. 1) The standard regimen dispensed is a percentage calculated by comparing the cumulative dose of each drug dispensed with the total recommended. Each drug received equal weight to a maximum of 100% per drug, as noted in the following formula for a three-drug regimen: percent standard regimen = $([D_1/SR_1] + [D_2/SR_2] + [D_3/SR_3]) \times (100/3)$, where $D_x$ is the cumulative dose dispensed of drug X and $SR_x$ is the recommended total dose. Patients with a score $\geq$80% were considered to have received an appropriate amount of antituberculosis medications (6). 2) The days without medication (identical to the "MED-OUT" adherence index validated for other medications) (7), for isoniazid or another drug required for the entire duration of treatment, is a percentage calculated by dividing the total number of days without medication (based on medication refill intervals and quantities dispensed) by the number of days between the first and last dispensing. The last refill does not influence this calculation. All the preceding calculations included all medicine dispensed to a patient, from all pharmacies required to report dispensing to the HMO to be reimbursed.

### Results

Medical records indicated that 27 (60%) of 45 TB cases were treated solely by HMO providers (Table 1); nearly all remaining patients received

Address for correspondence: Deborah Yokoe, 181 Longwood Ave., Boston, MA 02115, USA; fax: 617-731-1541; e-mail: deborah.yokoe@channing.harvard.edu.

Table 1. Characteristics of tuberculosis cases

| | Tuberculosis cases treated in HMO, n = 27 (60%) | Tuberculosis cases treated outside HMO, n = 18 (40%) |
|---|---|---|
| Mean age | 39 | 40 |
| Male | 16 (59%) | 7 (39%) |
| Foreign born | 22 (81%) | 15 (83%) |
| Pulmonary disease, with or without extrapulmonary involvement | 12 (44%) | 9 (50%) |
| Adequate prescribed regimen by treating physician | 26 (96%) | 17 (94%) |
| Antituberculosis medications dispensed through HMO pharmacies | 26 (96%) | 15 (83%) |
| Duration (in days) of antituberculosis medication dispensing by HMO (median, interquartile range) | 189 (148-291) | 1 (0-32) |
| Standard regimen dispensed by HMO (median, interquartile range) | 99% (86%-100%) | 24% (17%-40%) |

their non-HMO care in public health-funded TB programs. Thirty-seven (82%) cases received empiric regimens through pharmacies reimbursed by the HMO. In 34 (92%) instances, the empiric regimen dispensed was appropriate; for the remainder, empiric regimens contained too few drugs. Twenty-six (96%) of the 27 solely HMO-treated cases were prescribed a final antituberculosis regimen that was adequate in agents used, doses prescribed, and duration of treatment.

In 15 of the 18 cases treated at least partially outside the HMO, patients received some antituberculosis medication from pharmacies reimbursed by the HMO. In 14 of these cases, patients received medications only once or twice. A cutoff value of 70 days of drug dispensing through HMO-reimbursed pharmacies differentiated HMO-treated cases from cases treated in other settings. Among HMO-treated cases, 26 (96%) of 27 patients received medications for 70 days, compared with 1 (6%) of 18 who were at least partially treated outside the HMO (Figure 1) (RR = 17, 95% confidence

Figure 1. Pharmacy dispensing profiles of tuberculosis (TB) cases treated in the health maintenance organization (HMO) and outside the HMO. Standard regimen (percentage) and duration of dispensing of antituberculosis medication dispensed for TB cases. A cutoff value of ≥70 days of medication dispensed from HMO-reimbursed pharmacies, as assessed from automated pharmacy records, differentiated HMO-treated cases from cases at least partially treated outside the HMO.

interval [CI] 3 to 117, p <0.0001) (8). In HMO cases, median duration of dispensing from HMO pharmacies was 189 days (interquartile range: 148 days to 291 days) and the median standard regimen dispensed score was 99% (interquartile range: 86% to 100%). In cases outside the HMO, median duration of dispensing through HMO-reimbursed pharmacies was 1 day (interquartile range: 0 days to 32 days), and the median standard regimen dispensed score was 24% (interquartile range: 17% to 40%). Figure 2 shows the relationship between the appropriateness of the amount of medications dispensed and the timeliness of medication refills. In 4 (15%) of 26 HMO-treated cases, standard regimen dispensed scores were <80%, and days without medication scores were >30%. In only one of these four undertreated cases did the treating physician document nonadherence. Two other patients who received 100% of a standard regimen with unremarkable refill intervals were noted as noncompliant in physicians' medical records.

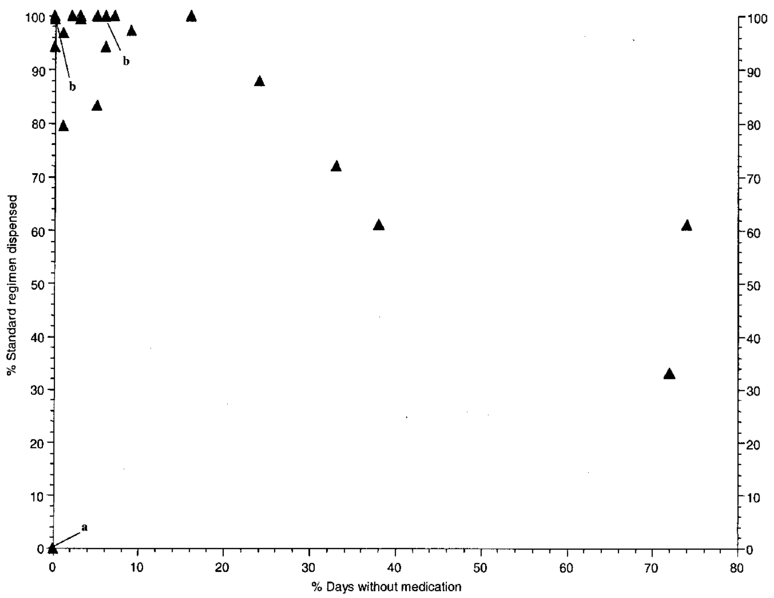

[a]In this case, no antituberculosis medications were dispensed through HMO pharmacies.

[b]In this case, the treating physician noted that the patient was nonadherent to prescribed medications.

Figure 2. Appropriateness of the amount of antituberculosis medications dispensed and the timeliness of medication refills. Percentage of standard regimen dispensed is plotted against percentage of days without antituberculosis medication for tuberculosis cases treated in the health maintenance organization.

### Conclusions

Automated pharmacy data provided useful information both about physicians' intended management of TB and about patients' adherence to prescribed therapy. The ability to monitor these aspects of TB care efficiently is particularly important when care is decentralized or a substantial proportion of patients receive care from more than one provider. We were able to determine that in nearly all cases appropriate initial or empiric regimens were prescribed, and that in most cases managed by HMO providers full ATS/CDC-recommended regimens were dispensed. This approach was thus more informative and efficient than prior study methods that assessed prescribed regimens by reviewing patients' medical records (9). Many HMOs and other insurers routinely monitor pharmacy dispensing records for various reasons. While the initial cost of creating a routine monitoring report varies, the marginal cost of running it periodically is usually negligible. This allows 100% surveillance of therapy, compared with manual record review, which is more expensive even when only a sample of records is reviewed. Additional investigation would determine whether this method will be helpful for other infectious diseases, such as pelvic inflammatory disease.

In using a pharmacy-based system to monitor adherence to therapy, it is important to identify cases treated solely within a delivery system, since automated pharmacy information is reliably complete only for these persons. Restricting to patients with at least 70 days of therapy accomplishes this, since it excludes those who receive empiric regimens within one health system and then complete their care at another. Most care delivered by non-HMO providers was provided by public health clinics. Potential physician incentives for transfer of care included closer monitoring of therapy by experts in TB treatment, particularly in difficult-to-manage cases. Patients' incentives included free medications provided by the department of public health. Although all patients described here had pharmacy benefits, the cost to the patients of copayments for a standard treatment regimen would have been $75 to $200.

Monitoring automated pharmacy records of patients treated for TB was an efficient adjunct for monitoring adherence but did not entirely replace providers' assessments documented in the medical records. It also provided no advantage in settings that used directly observed therapy, which is not routinely used in Massachusetts.

Pharmacy records may be used to contribute to management of TB in two ways: in real time, to identify suboptimal regimens and noncompliant patients and periodically, to assess overall appropriateness of care. In real time, one might monitor the dispensing of antituberculosis medications for confirmed cases of TB, intervening if necessary with physicians to ensure that appropriate regimens are used and with patients to minimize gaps in dispensing. Identifying noncompliant patients in a regular and timely manner could allow for interventions (e.g., directly observed therapy) to improve adherence to the treatment regimen. Such oversight could be coordinated with or overseen by public health agencies. Coordination between delivery systems and public health agencies is expected to become an important element of TB control (10). Periodic assessment of dispensing records can also provide a simple, efficient measure of the overall appropriateness of TB care in a wide array of settings. These measures would allow targeting of resources to improve organizations' management of TB. If these findings are confirmed in other settings, routine monitoring of dispensing of antituberculosis medications may be useful as an adjunct to other methods of assessing and ensuring appropriate therapy.

### Acknowledgment

We thank Claire Canning for her assistance with this study.

This study was supported by CDC Contract 200-95-0957-010 and the Harvard Pilgrim Health Care Foundation.

Dr. Subramanyan was a research fellow at the Channing Laboratory, Brigham and Women's Hospital, Boston, Massachusetts, and a student at Harvard Medical School when he performed this work. He is currently a resident at the University of California, San Francisco.

### References

1. Yokoe DS, Subramanyan GS, Nardell E, Sharnprapai S, McCray E, Platt R. Supplementing tuberculosis surveillance with automated data from health maintenance organizations. Emerg Infect Dis 1999;5:779-87.
2. Subramanyan GS, Yokoe DS, Sharnprapai S, Tang Y, Platt R. An algorithm to match registries with minimal disclosure of individual identities. Public Health Rep 1999;114:91-3.
3. Centers for Disease Control and Prevention. Case definitions for infectious conditions under public health surveillance. MMWR Morb Mortal Wkly Rep 1997;46:40-1.
4. Bass JB Jr, Farer LS, Hopewell PC, O'Brien R, Jacobs RF, Ruben F, et al. Treatment of tuberculosis and tuberculosis infection in adults and children. Am J Respir Crit Care Med 1994;149:1359-74.
5. American Thoracic Society. Treatment of tuberculosis and tuberculosis infection in adults and children. American Review of Respiratory Disease 1986;134:355-63.
6. Fox W. Whither short-course chemotherapy? British Journal of Diseases of the Chest 1981;75:331-57.
7. Steiner JF, Koepsell TD, Fihn SD, Inui TS. A general method of compliance assessment using centralized pharmacy records: description and validation. Med Care 1988;26:814-23.
8. EpiInfo [computer program]. Version 6.04b. Atlanta (GA): Centers for Disease Control and Prevention; 1997.
9. Migliori GB, Spanevello A, Ambrosetti M, Neri M. Surveillance of tuberculosis treatment prescriptions in Italy. The Varese TB Study Group. Monaldi Arch Chest Dis 1998;53:37-42.
10. Halverson PK, Mays GP, Miller CA, Kaluzny AD, Richards TB. Managed care and the public health challenge of TB. Public Health Rep 1997;112:22-8.
